# Supplementary material for: Diagnostic accuracy of cytology for the detection of endometrial cancer in urine and vaginal samples
Source: Nat Commun. 2021 Feb 11;12:952. doi: 10.1038/s41467-021-21257-6 (PMC7878864; doi:10.1038/s41467-021-21257-6)
Supplement: Supplementary file 1 — Reporting Summary [file 41467_2021_21257_MOESM1_ESM.pdf]

nature research

Corresponding author(s): Emma Crosbie

Last updated by author(s): Jan 5, 2021

Reporting Summary

Nature Research wishes to improve the reproducibility of the work that we publish. This form provides structure for consistency and transparency in reporting. For further information on Nature Research policies, see our Editorial Policies and the Editorial Policy Checklist.

Statistics

For all statistical analyses, confirm that the following items are present in the figure legend, table legend, main text, or Methods section.

n/a	Confirmed	
☐	☒	The exact sample size (n) for each experimental group/condition, given as a discrete number and unit of measurement
☐	☒	A statement on whether measurements were taken from distinct samples or whether the same sample was measured repeatedly
☐	☒	The statistical test(s) used AND whether they are one- or two-sided<br>*Only common tests should be described solely by name; describe more complex techniques in the Methods section.*
☒	☐	A description of all covariates tested
☒	☐	A description of any assumptions or corrections, such as tests of normality and adjustment for multiple comparisons
☐	☒	A full description of the statistical parameters including central tendency (e.g. means) or other basic estimates (e.g. regression coefficient) AND variation (e.g. standard deviation) or associated estimates of uncertainty (e.g. confidence intervals)
☐	☒	For null hypothesis testing, the test statistic (e.g. F, t, r) with confidence intervals, effect sizes, degrees of freedom and P value noted<br>*Give P values as exact values whenever suitable.*
☒	☐	For Bayesian analysis, information on the choice of priors and Markov chain Monte Carlo settings
☒	☐	For hierarchical and complex designs, identification of the appropriate level for tests and full reporting of outcomes
☒	☐	Estimates of effect sizes (e.g. Cohen's d, Pearson's r), indicating how they were calculated

Our web collection on statistics for biologists contains articles on many of the points above.

Software and code

Policy information about availability of computer code

Data collection	No software was used.
Data analysis	Results were analysed using SPSS (version 22 IBM).

For manuscripts utilizing custom algorithms or software that are central to the research but not yet described in published literature, software must be made available to editors and reviewers. We strongly encourage code deposition in a community repository (e.g. GitHub). See the Nature Research guidelines for submitting code & software for further information.

Data

Policy information about availability of data

All manuscripts must include a data availability statement. This statement should provide the following information, where applicable:

- Accession codes, unique identifiers, or web links for publicly available datasets
- A list of figures that have associated raw data
- A description of any restrictions on data availability

All data are available within the Article, Source Data or available from the corresponding author upon request.

Field-specific reporting

Life sciences study design

All studies must disclose on these points even when the disclosure is negative.

Sample size	A power calculation found that a sample size of 100 women with endometrial cancer and 100 women with post-menopausal bleeding would enable the diagnostic accuracy of cytology to be estimated to +/-7%, assuming sensitivity and specificity of ~85%. It was based on an estimated 5% endometrial cancer prevalence in women presenting to our service with post-menopausal bleeding. An assumed sensitivity and specificity of 85% was considered the minimum acceptable diagnostic accuracy at which the test might be taken forward for validation. The +/-7% relates to the 95% confidence interval surrounding our estimated sensitivity/ specificity.
Data exclusions	Data were excluded only where samples were missing or considered inadequate for cytological review.
Replication	Samples were analysed by two members of the cytology team and reviewed by a third in case of discrepancies.
Randomization	Not applicable to the study because participants were not assigned to different interventions.
Blinding	Researchers were blinded to participant cancer status at the time of sample acquisition for those women in the prospective post-menopausal bleeding cohort. Researchers were aware of participant cancer status in the known/ suspected cancer cohort at the time of sample acquisition. Cytopathologists were blinded to participant cancer status throughout sample preparation, analysis and reporting until they had produced their final, consensus cytology report.

Reporting for specific materials, systems and methods

We require information from authors about some types of materials, experimental systems and methods used in many studies. Here, indicate whether each material, system or method listed is relevant to your study. If you are not sure if a list item applies to your research, read the appropriate section before selecting a response.

Materials & experimental systems

n/a	Involved in the study	
☒	☐	Antibodies
☒	☐	Eukaryotic cell lines
☒	☐	Palaeontology and archaeology
☒	☐	Animals and other organisms
☐	☒	Human research participants
☒	☐	Clinical data
☒	☐	Dual use research of concern

Methods

n/a	Involved in the study	
☒	☐	ChIP-seq
☒	☐	Flow cytometry
☒	☐	MRI-based neuroimaging

Human research participants

Policy information about studies involving human research participants

Population characteristics	The population characteristics are fully described in Table 1 of the Article.
Recruitment	We recruited women attending the Gynaecology Outpatient Department at St Mary's Hospital, Manchester University NHS Foundation Trust (MFT) between June-2016 and October-2018. Women referred for urgent investigation of unexplained post-menopausal bleeding and those with known or suspected endometrial cancer awaiting hysterectomy were eligible to take part. We excluded women with unexplained abnormal pre-menopausal bleeding as well as those who had previously had a hysterectomy. There was minimal self-selection bias because >95% of invited women agreed to participate in the study.
Ethics oversight	North-West Greater Manchester Research Ethics Committee (reference-16/NW/0660)

Note that full information on the approval of the study protocol must also be provided in the manuscript.
